# Risk factors for neonatal catheter-related bloodstream infections: a systematic review and meta-analysis

**DOI:** 10.3389/fpubh.2025.1719016

**Published:** 2025-12-19

**Authors:** Shuai Wang, Lihong Chi, Xingye Zhou

**Affiliations:** 1 Outpatient Department, Hainan Women and Children's Medical Center, Haikou, Hainan, China; 2 Infection Control Department, Hainan Women and Children's Medical Center, Haikou, Hainan, China; 3 Hospital Infection-Control Department, The Second Affiliated Hospital of Hainan Medical University, Haikou, Hainan, China; 4 School of Public Health, Hainan Medical University, Haikou, Hainan, China

**Keywords:** neonate, catheter-related bloodstream infection, CRBSI, risk factor, meta-analysis

## Abstract

**Background:**

Catheter-related bloodstream infection (CRBSI) is a prevalent nosocomial infection in neonatal units. The incidence of CRBSI can prolong hospitalization, cause irreparable harm, and negatively affect newborn survival and quality of life. Previous research has identified risk factors for CRBSI, but the findings have been inconsistent, and all predisposing factors have not been systematically described. This study aimed to investigate the risk factors for developing CRBSI in neonates and to provide a scientific basis for decision-making in the prevention of neonatal CRBSI.

**Methods:**

A systematic search of PubMed, Web of Science, Scopus, China National Knowledge Infrastructure (CNKI), and the Wanfang Database was performed from the time of each database’s inception to 1 October 2025. The search strategy combined subject terms and free-text keywords. The Newcastle–Ottawa scale (NOS) was used to assess the quality of the literature, and meta-analysis was carried out using RevMan 5.3. Heterogeneity was evaluated using the *I^2^* statistic method, and publication bias was analyzed with funnel plot tests.

**Results:**

A total of 18 articles involving 11,963 participants were included in this study. In the meta-analysis, the risk factors for the development of CRBSI in neonates, ranked from strongest to weakest association, were as follows: Multi-lumen central venous catheters (CVCs) ≥ 2, 5-min Apgar score≤7, number of manipulations≥2, catheter indwelling time>14d, gestational age≤32w, parenteral nutrition (PN), maternal disease, birth weight<1,500 g, male sex, and catheterization of the upper and lower limbs. These risk factors were found to be significantly associated with the development of CRBSI.

**Conclusion:**

This meta-analysis identifies key modifiable risk factors for CRBSI in neonates, informing a proposed evidence-based prevention bundle. This bundle targets factors such as catheter dwell time, aseptic technique, and nutrition management to reduce CRBSI incidence and improve resource efficiency, especially in high-risk neonates.

## 1 Introduction

With the rapid advancement of neonatal diagnostic and therapeutic technologies, arteriovenous catheterization has become a crucial pathway for neonates, especially those with low birth weight (1). Arteriovenous catheterization is viewed as an invasive technique. If the catheter is punctured too frequently (2) or left in place for an extended period of time (3), it can cause local or systemic infections and serve as an entry point for catheter-related bloodstream infection (CRBSI). CRBSI is one of the most common nosocomial infections and has been linked to increased neonatal morbidity, mortality, and hospitalization costs (4, 5). It remains a major challenge worldwide, particularly in developing nations (6), and has attracted widespread attention.

There are several factors that contribute to the significantly increased risk of CRBSI in neonates. These include their immature immune systems, fragile skin, frequent exposure to invasive procedures and medical devices, and variability in healthcare personnel practices (7). This is particularly true for high-risk infants in intensive care units. The incidence of CRBSI in NICUs in high-income European nations has been found to be 1.8–8.4 per 1,000 catheter-days (8). In contrast, a large-scale study in India reported an overall CRBSI incidence in NICUs as high as 36.2 per 1,000 catheter-days (9), a situation of great concern. It is urgent to actively explore the risk factors for CRBSI in neonates and to implement effective prevention strategies.

In 2023, a systematic review assessed the risk factors for CRBSI in patients aged 18 years and older (10). It found that total parenteral nutrition (PN), multi-lumen devices, chemotherapy, immunosuppression, and duration of catheterization may increase the incidence of CRBSI. Neonates represent a uniquely vulnerable population owing to immature immune systems, distinct catheter types (e.g., umbilical venous catheters), and specific care practices, which may differentiate their CRBSI risk factors from those observed in adults or mixed-age groups. However, the study did not investigate factors such as sex, number of punctures, or birth weight, and the study population did not include neonates. To date, no systematic reviews focusing specifically on CRBSI in newborns have been published. Therefore, we performed a systematic review and meta-analysis to investigate the risk factors for CRBSI in neonates and to offer a theoretical basis for its prevention.

## 2 Materials and methods

Our systematic review protocol was registered with the International Prospective Register of Systematic Reviews (PROSPERO; registration number CRD42024600356).

### 2.1 Search strategy

Relevant studies published from the inception of each database to 1 October 2025 were searched in PubMed, Web of Science, Scopus, China National Knowledge Infrastructure (CNKI), and the Wanfang databases. No language restrictions were applied. The search strategy was developed by combining controlled descriptors from each database with the keywords: “Catheter-related bloodstream infection,” “Neonatal,” and “Risk factors.” The complete search strategy is presented in Supplementary material 1.

### 2.2 Inclusion and exclusion criteria

We applied the following criteria to select eligible studies. The inclusion criteria were as follows: (1) Study design was randomized control trials, cohort studies, or case–control studies; (2) study participants were neonates with indwelling central venous catheters, aged ≤28 days; (3) risk factors associated with CRBSI were reported; (4) catheter-associated bacteremia was defined according to the criteria of the Centers for Disease Control and Prevention (CDC)/National Healthcare Safety Network (NHSN); (5) reported relative risk (RR) or odds ratios (ORs) with corresponding 95% confidence intervals (CIs) for CRBSI; and (6) the primary outcome was a laboratory-confirmed bloodstream infection meeting standard diagnostic criteria for CRBSI or CLABSI (6). While these terms reflect different clinical and surveillance applications, both encompass infections occurring during catheterization or within 48 h of catheter removal. Given our focus on identifying catheter-specific risk factors to inform clinical diagnosis and etiology, we employed the more rigorously defined CRBSI criteria throughout this study.

The exclusion criteria were as follows: (1) Study participants were not neonates; (2) studies not focused solely on CRBSI; (3) studies not addressing risk factors; (4) case reports, reviews, conference papers, patents, and duplicate publications; (5) studies with inaccessible information material, errors, missing data, or low quality; (6) studies with no control group; and (7) unpublished reports and gray literature.

### 2.3 Data extraction

Data extraction from the included primary studies was performed by two independent reviewers, and any disagreements were resolved through discussion and consensus with a third reviewer. For each study, the following information was extracted: authors, year of study publication, country, study design, study population, number of cases and controls, and statistically significant findings from univariate or multivariate analysis. We preferred multivariate risk ratios (RRs)/ORs with corresponding 95% confidence intervals (CIs) rather than univariate results.

### 2.4 Quality assessment

The Newcastle–Ottawa scale (NOS) (11) was used to assess the quality of evidence of the included studies. High-quality studies scored 8–9, moderate-quality studies scored 6–7, and low-quality studies scored <6. Furthermore, two authors independently assessed the quality of each study in this meta-analysis, and disagreements were resolved through discussion. Disagreements were resolved by consulting a third reviewer.

### 2.5 Statistical analysis

We used the RevMan 5.3 software to perform a meta-analysis of the compliance outcome indicators. The magnitude of heterogeneity: if *p* > 0.1, *I^2^* < 50% was considered low homogeneity, and a fixed-effects model was selected; if *p* ≤ 0.1, *I^2^* ≥ 50% was considered high heterogeneity, and a random-effects model was selected (12). Forest plots were also generated to illustrate the results of the combined analysis. Sensitivity analysis and subgroup analysis were performed to find the source of heterogeneity for studies exhibiting substantial heterogeneity. Funnel plots (13) and Egger’s test (14) were used to measure publication bias. Combined odds ratios (ORs) with corresponding 95% CIs were calculated to assess the risk factors for CRBSI. The reported probability values were two-sided, and a *p*-value of ≤0.05 was considered statistically significant.

## 3 Results

### 3.1 Study selection

**Figure 1:**
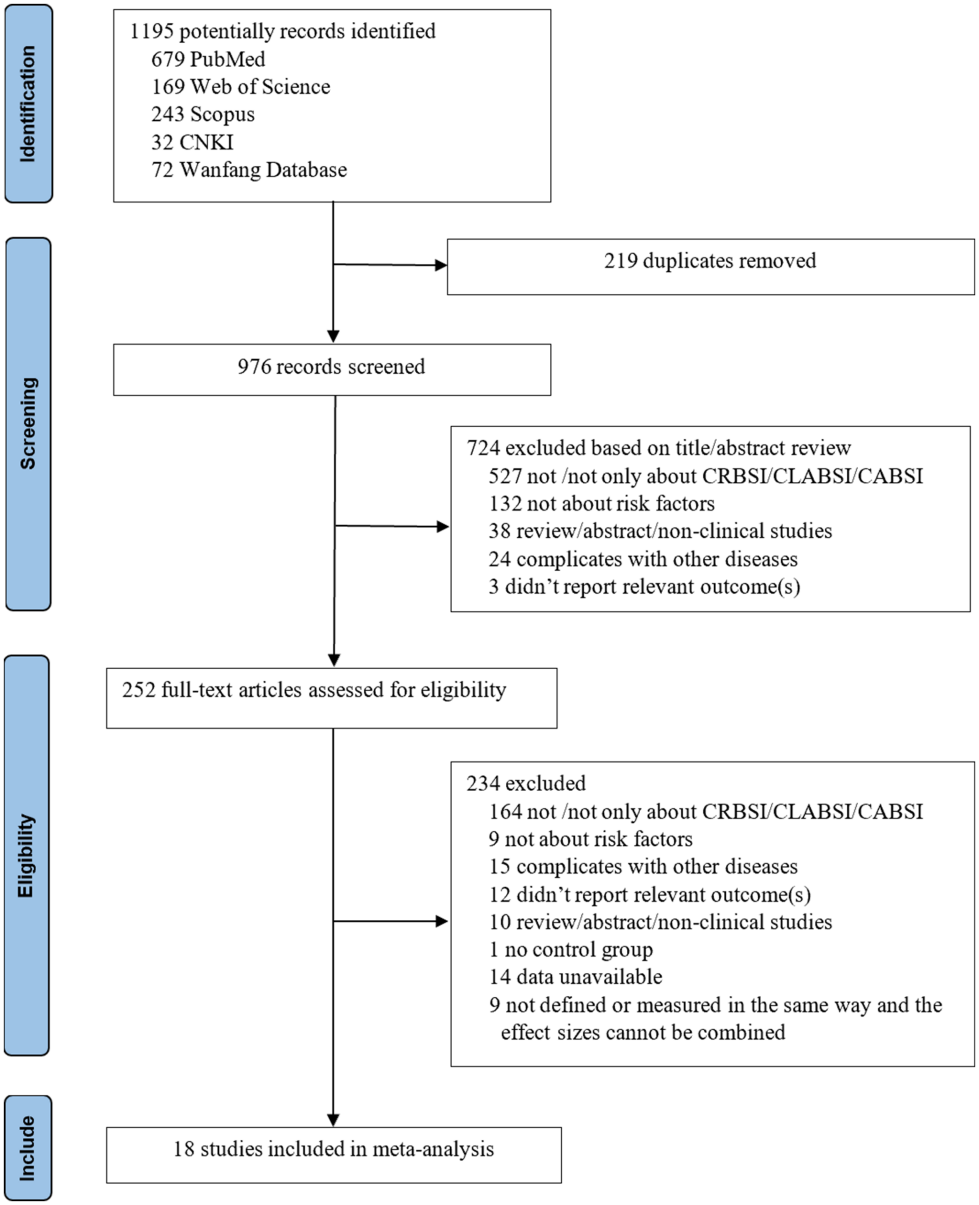
Flow diagram illustrating the selection and disposition of studies.

Using the described search strategy, we identified a total of 1,195 studies, of which 219 were excluded for duplication. Based on the inclusion and exclusion criteria, we further excluded 724 studies, leaving 252 studies for full-text review. After reviewing the full texts, 234 studies were excluded. Ultimately, 18 studies were included in the meta-analysis. The flowchart for study selection is shown in Figure 1.

### 3.2 Study characteristics

The characteristics of the included studies are shown in Table 1. The studies included in the qualitative synthesis were 12 case–control studies (15–26) and six cohort studies (27–32), published between 2011 and 2024. The countries represented in this study included China (15–23, 25), Mexico (24), the United States of America (27), South Africa (26), the Netherlands (28), Indonesia (29), Brazil (30), Switzerland (31), and Thailand (32). A total of 11,963 participants were included in this meta-analysis, including 860 cases and 11,103 controls.

**Table 1:** Characteristics of the studies included.

Author (year)	Country	Study design	Case/control, n	Male sex, case/control, n	Population	Vascular access device	Outcomes of interest	Multivariate	Quality rating/score
Li X et al. (2022) (15)	China	Case–control	40/777	9/281	NICU	PICC	Nation Birth weight Catheter dwell time Number of manipulations	Birth weight(OR = 5.218, 2.861–9.518) Number of manipulations(OR = 16.523, 5.548–49.212)	7
Lv Q et al. (2019) (16)	China	Case–control	53/371	31/269	Normal	PICC	Birth weight≤1,500 g Catheter dwell time≥20 d 5-min Apgar score≤7	Birth weight≤1,500 g(OR = 4.105, 2.067–8.155) Catheter dwell time≥20 d(OR = 9.561, 4.573–19.986) 5-min Apgar score≤7(OR = 4.381, 2.172–8.836)	7
Li CH et al. (2014) (17)	China	Case–control	18/132	15/100	Normal	PICC	Birth weight≤1,500 g Gestational age≤32w 5-min Apgar score≤7 Catheter dwell time Respiratory distress syndrome	Birth weight (OR = 2.632, 2.125–2.854) Gestational age≤32w (OR = 3.254, 3.148–4.152) Catheter dwell time (OR = 6.541, 3.125–7.128) 5-min Apgar score≤7 (OR = 25.214, 5.254–174.589)	6
Huang CM et al. (2013) (18)	China	Case–control	13/167	11/110	Normal	PICC	Gestational age≤32w Birth weight≤1,500 g Male sex 5-min Apgar score≤7 Respiratory distress syndrome	Birth weight (OR = 2.216, 2.108–2.899) Male sex (OR = 4.011, 1.365–23.941) 5-min Apgar score≤7(OR = 23.316, 3.882–198.416)	7
Chen ZH. (2023a) (19)	China	Case–control	49/334	29/218	Normal	PICC	Birth weight Delivery modes Catheter dwell time Number of manipulations Manipulate time Catheter blockage Maternal disease factor	Delivery modes(Cesarean section)(OR = 9.043, 2.697–30.328) Catheter dwell time(OR = 4.186, 2.304–7.606) Number of manipulations(OR = 3.033, 1.507–6.102) Manipulate time(OR = 7.257, 3.349–15.728) Maternal disease factor(OR = 5.401, 1.633–17.866)	7
Chen ZH et al. (2023b) (20)	China	Case–control	20/61	12/40	Normal	PICC	Gestational age Age at catheter insertion Reactive protein at catheterization Diabetes mellitus Meconium-stained amniotic fluid Catheter dwell time	Gestational age(OR = 2.451, 1.856–2.956) Age at catheter insertion(OR = 3.021, 2.402–3.512) Reactive protein at catheterization(OR = 2.285, 1.625–2.815) Diabetes mellitus(OR = 2.815, 1.925–3.412) Meconium-stained amniotic fluid(OR = 2.486, 2.021–2.956) Catheter dwell time(OR = 2.698, 2.184–3.241)	6
Xu HL et al. (2022) (21)	China	Case–control	77/650	—	Normal	PICC	Birth weight 5-min Apgar score Catheter dwell time Catheter blockage	Birth weight(OR = 1.852, 1.100–2.998) 5-min Apgar score (OR = 2.499, 1.297–4.815) Catheter dwell time(OR = 1.970, 1.152–3.370) Catheter blockage(OR = 2.821, 1.720–4.626)	7
Zhang Y et al. (2024) (22)	China	Case–control	38/642	31/486	NICU	PICC	Gestational age (w) Birth weight (g) Nutrition support mode (cases) Congenital diseases (cases) Catheterization time Respiratory distress syndrome 5-min Apgar score ≤ 7	Gestational age (OR = 3.267, 3.157–4.160) Birth weight (OR = 2.598, 2.064–2.772) Catheterization time (OR = 6.488, 3.143–7.268) 5-min Apgar score ≤ 7 (OR = 24.855, 5.127–175.603)	8
Hu Y et al. (2021) (23)	China	Case–control	41/345	24/211	NICU	PICC	Birth weight Duration of PICC stay 5-min Apgar score Site of PICC insertion	Birth weight ≤ 1,500 g (OR = 1.923, 1.135–2.629) Duration of PICC stay ≥ 21 days (OR = 2.077, 1.024–2.431) 5-min Apgar score ≤ 7 (OR = 2.198, 1.135–3.414) Femoral vein PICC insertion (OR = 3.044, 1.989–4.306)	7
Garcia H et al. (2019) (24)	Mexico	Case–control	74/105	41/61	NICU	CVC	Age at CVC insertion (days) Total NICU stay (days) Abdominal surgery Placement site of CVC: Internal jugular Placement site of CVC: Saphenous Placement site of CVC: Upper limb (Percutaneous insertion) Placement site of CVC: Brachial (Percutaneous insertion)	Abdominal surgery (OR = 2.6, 1.3–5.1) Length of hospitalization (≥14 d) (OR = 3.3, 1.5–6.9) Double lumen CVC (OR = 10.0, 2.3–44.3) Surgical cut-down technique (OR = 2.1, 1.1–4.1) CVC site (internal jugular vein) (OR = 2.7, 1.5–5.1) Dressing type (gauze and surgical tape) (OR = 2.1, 1.2–4.1) Blood transfusions (OR = 3.6, 1.87–6.78) Parenteral nutrition (OR = 3.3, 1.53–7.27) Number of CVC manipulations (>200) (OR = 2.7, 1.5–5.0) CVC indwelling total time (>21 d) (OR = 2.9, 1.5–5.4)	7
Xu YP et al. (2022) (25)	China	Case–control	50/396	35/211	NICU	PICC	Male sex Birth weight Gestational age Site of PICC insertion Use of fluconazole lock Duration of antibiotic use Catheter dwell time Length of hospital stay	Duration of antibiotic use (OR = 1.013, 1.005–1.020) Site of PICC insertion(upper and lower limbs; OR = 1.658, 1.060–2.593) Catheter dwell time (OR = 1.037, 1.009–1.065)	7
Milstone et al. (2013) (27)	USA	Retrospective cohort study	149/4648	—	NICU	PICC	Birth weight, 100 g (every 100-g increase in birth weight) Concurrent PICCs	Concurrent PICCs (OR = 2.04, 1.12–3.71)	8
Geldenhuys et al. (2017) (26)	South African	Case–control	19/76	13/48	NICU	UVC, PICC, CVC, and Broviac	Gestational age (weeks) Birth weight (g) Length of NICU stay (days) Catheter dwell time in NICU Type of central line (UVC) Insertion venue Surgery during NICU stay	Length of stay >30 days (OR = 20.7, 2.1–203.2) Insertion venue: Theater (OR = 17.5, 3.1–97.1)	8
Dubbink et al. (2017) (28)	Netherlands	Descriptive cohort study	35/621	27/335	NICU	FVC, UVC, and PICC	Gestational age at birth (weeks) Birth weight (g) Cyanotic congenital heart disease (CHD, including asphyxia requiring therapeutic cooling was indicated) Duration of hospitalization (days) Catheter-days Age at catheter insertion Age at catheter removal Antibiotic treatment within 24 h postpartum Parenteral nutrition during catheter dwell time	Parenteral nutrition (HR = 2.60, 1.25–5.41) Male sex (HR = 2.63, 1.17–5.90) Higher birth weight (HR = 1.04, 1.002–1.09)	8
Ekaputri et al. (2022) (29)	Indonesia	Prospective cohort study	31/83	_	Normal	PICC	Dwell time	PICC Dwell time >14 days (RR = 2.988, 1.191–7.497)	7
Sarmento et al. (2023) (30)	Brazil	Prospective cohort study	39/242	_	NICU	PICC	Location of puncture	Location of puncture: Upper limbs (RR = 2.84, 1.02–6.85)	7
Zingg et al. (2011) (31)	Switzerland	Prospective cohort study	102/1022	_	NICU、PICU	PICC, UAC, and UVC	Extremely low birth weight Pluriparity Apgar score Parenteral nutrition Antibiotic use	Extremely low birth weight (HR = 2.24, 1.42–3.53) Parenteral nutrition (HR = 2.23, 1.10–4.55)	8
Khieosanuk et al. (2022) (32)	Thailand	Retrospective cohort study	12/431	_	Normal	CVC	Congenital heart defects requiring surgery Other underlying medical conditions Type of CVC: Short-term non-tunneled catheter Triple-lumen CVC (vs. single-lumen CVC) Place of CVC insertion: Operating room Insertion vessel Umbilical vein/artery CVC inserter: Anesthesiologist/surgeon	Type of CVC: Triple-lumen CVC (vs. single-lumen CVC) (OR = 8.33, 1.29–53.79)	7

OR, Odds ratio; RR, Relative risk; HR, Hazard ratio; CI, Confidence interval; PICC, Peripheral insertion central catheter; UVC, Umbilical venous catheter; CVC, Central venous catheter; FVC, Femoral venous catheters; UAC, Umbilical artery catheters; NICU, Neonatal intensive Care Unit; Normal, Routine inpatient ward. “a” and “b” denote distinct articles authored by the same individual within the same calendar year. USA, United States of America.

### 3.3 Quality assessment

The quality of the included cohort studies, as assessed using the Newcastle-Ottawa Scale, was generally moderate to high. The majority of studies secured a score of 7 or higher out of 9. In addition, most studies demonstrated adequate selection of cohorts and comparability. The most common potential source of bias was in the ‘Outcome’ domain, where some studies did not explicitly state whether the assessment of CRBSI was conducted blinded to exposure status (Tables 2, 3). Consequently, based on the quality assessment of the included studies, the results of our meta-analysis are considered reliable, with an overall moderate to high study quality and a relatively low risk of bias.

**Table 2:** Methodological quality of the included case–control studies based on the Newcastle–Ottawa scale (NOS).

Included studies	Year	Is the definition adequate?	Representativeness of cases	Selection of controls	Definition of controls	Comparability of both groups	Ascertainment of diagnosis	Same ascertainment method for both groups	Non-response rate	Total scores
Li X	2022	*	*		*	**	*	*		7
Lv Q	2019	*	*		*	**	*	*		7
Li CH	2014	*	*		*	*	*	*		6
Huang CM	2013	*	*		*	**	*	*		7
Chen ZH	2023a	*	*		*	**	*	*		7
Chen ZH	2023b	*	*		*	*	*	*		6
Xu HL	2022	*	*		*	**	*	*		7
Zhang Y	2024	*	*		*	**	*	*	*	8
Hu Y	2021	*	*		*	**	*	*		7
Garcia H	2019	*	*		*	**	*	*		7
Xu YP	2022	*	*		*	**	*	*		7
Geldenhuys	2017	*	*		*	**	*	*	*	8

This scale uses asterisks (*) as the scoring unit, with a total score of 9 stars. The maximum score for each item is 1 star, but in the comparability module, the maximum score can reach 2 stars. “a” and “b” denote distinct articles authored by the same individual within the same calendar year.

**Table 3:** Methodological quality of the included cohort studies based on the Newcastle–Ottawa scale (NOS).

Included studies	Year	Representativeness of the exposed cohort	Selection of the non-exposed cohort	Ascertainment of exposure	Demonstration that outcome of interest was not present at the start of the study	Comparability of cohorts on the basis of the design or analysis	Assessment of outcomes	Was follow-up long enough for outcomes to occur	Adequacy of cohort follow-up	Total scores
Milstone	2013	*	*	*	*	**	*	*		8
Dubbink	2017		*	*	*	**	*	*	*	8
Ekaputri	2022	*	*	*	*	*	*	*		7
Sarmento	2023		*	*	*	**	*	*		7
Zingg	2011		*	*	*	**	*	*	*	8
Khieosanuk	2022		*	*	*	**	*	*		7

This scale uses asterisks (*) as the scoring unit, with a total score of 9 stars. The maximum score for each item is 1 star, but in the comparability module, the maximum score can reach 2 stars.

### 3.4 Risk factors for CRBSI

Initially, we identified 19 risk factors from the 18 included studies. Of these, nine factors could not be quantitatively analyzed in the present study due to (1) insufficient data; (2) remaining risk factors not being defined or measured consistently across studies, preventing conclusive statistical analysis; (3) being analyzed in only one study; and (4) non-significant results in the analyses performed. Thus, 10 risk factors reported in at least two studies remained for quantitative analysis.

#### 3.4.1 Multi-lumen CVCs ≥ 2

**Figure 2:**
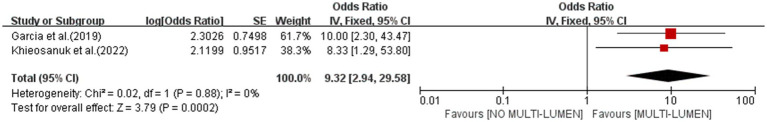
The forest plot shows the relationship between multi-lumen CVCs≥2 and the risk of CRBSI.

A meta-analysis of two studies showed that multi-lumen central venous catheters (CVCs) ≥ 2 were associated with the risk of CRBSI (OR = 9.32; 95% *CI*: 2.94–29.58; *I^2^* = 0%; Figure 2).

#### 3.4.2 5-min Apgar score≤7

**Figure 3:**
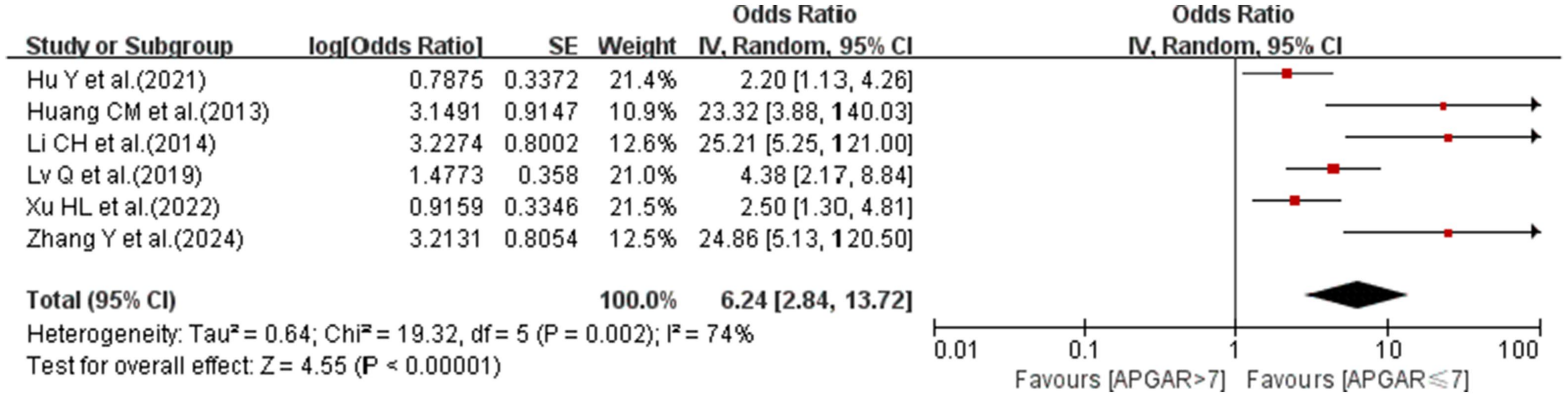
The forest plot shows the relationship between a 5-min Apgar score (APGAR) ≤ 7 and the risk of CRBSI.

A meta-analysis of six studies showed that a 5-min Apgar score≤7 was associated with the risk of CRBSI (OR = 6.24; 95% *CI*: 2.84–13.72; *I^2^* = 74%; Figure 3).

#### 3.4.3 Number of manipulations≥2

**Figure 4:**
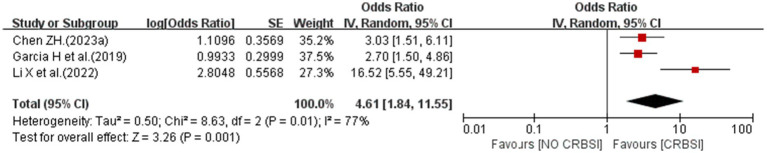
The forest plot shows the relationship between the number of manipulations≥2 and the risk of CRBSI.

A meta-analysis of three studies showed that a number of manipulations≥2 was associated with the risk of CRBSI (OR = 4.61; 95% *CI*: 1.84–11.55; *I^2^* = 77%; Figure 4).

#### 3.4.4 Catheter indwelling time>14d

**Figure 5:**
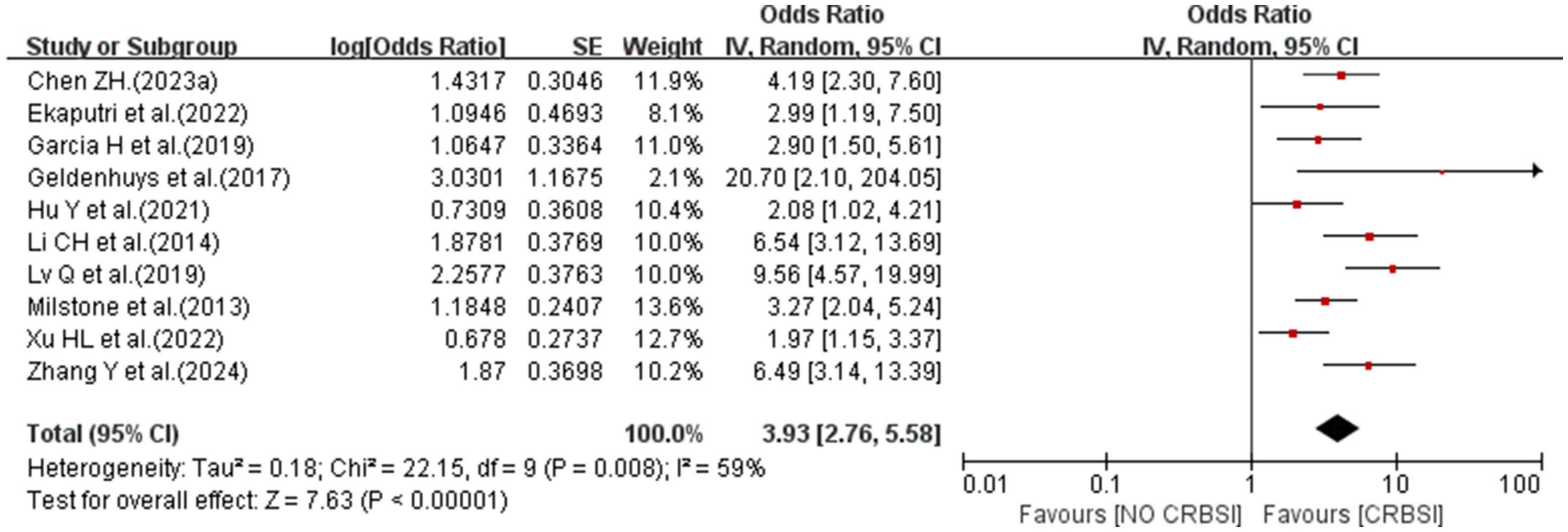
The forest plot shows the relationship between catheter indwelling time>14d and the risk of CRBSI.

A meta-analysis of 10 studies showed that catheter indwelling time>14d was associated with the risk of CRBSI (OR = 3.93; 95% *CI*: 2.76–5.58; *I^2^* = 59%; Figure 5).

#### 3.4.5 Gestational age≤32w

**Figure 6:**
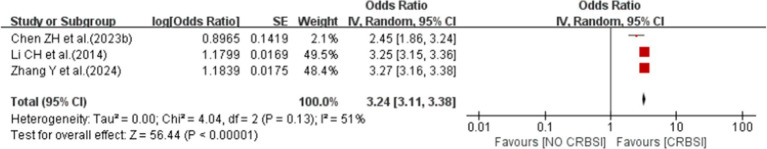
The forest plot shows the relationship between gestational age ≤32w and the risk of CRBSI.

A meta-analysis of three studies showed that gestational age≤32w was associated with the risk of CRBSI (OR = 3.24; 95% *CI*: 3.11–3.38; *I^2^* = 51%; Figure 6).

#### 3.4.6 Parenteral nutrition

**Figure 7:**
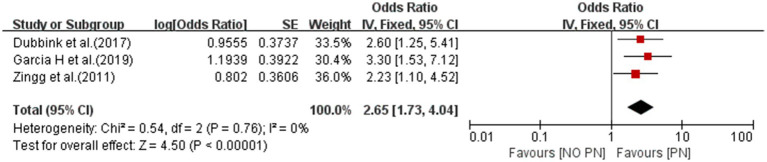
The forest plot shows the relationship between parenteral nutrition (PN) and the risk of CRBSI.

A meta-analysis of three studies showed that parenteral nutrition was associated with the risk of CRBSI (OR = 2.65; 95% *CI*: 1.73–4.04; *I^2^* = 0%; Figure 7).

#### 3.4.7 Maternal disease

**Figure 8:**
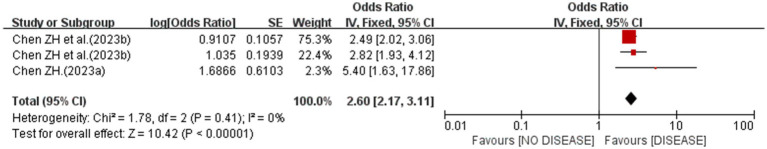
The forest plot shows the relationship between maternal disease and the risk of CRBSI.

A meta-analysis of three studies showed that maternal disease was associated with the risk of CRBSI (OR = 2.60; 95% *CI*: 2.17–3.11; *I^2^* = 0%; Figure 8).

#### 3.4.8 Birth weight<1,500 g

**Figure 9:**
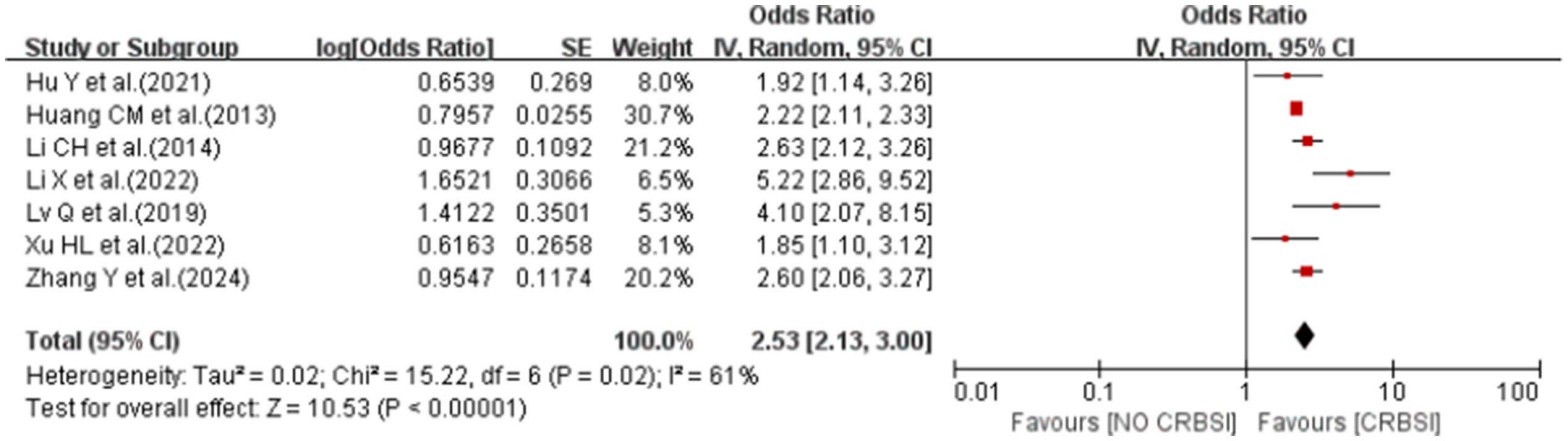
The forest plot shows the relationship between birth weight<1,500 g and the risk of CRBSI.

A meta-analysis of seven studies showed that birth weight<1,500 g was associated with the risk of CRBSI (OR = 2.53; 95% *CI*: 2.13–3.00; *I^2^* = 61%; Figure 9).

#### 3.4.9 Male sex

**Figure 10:**
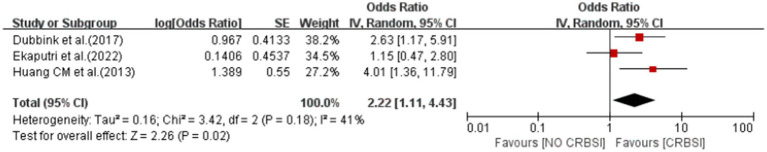
The forest plot shows the relationship between male sex and the risk of CRBSI.

A meta-analysis of three studies showed that male sex was associated with the risk of CRBSI (OR = 2.18; 95% *CI*: 1.29–3.68; *I^2^* = 41%; Figure 10).

#### 3.4.10 Catheterization of the upper and lower limbs

**Figure 11:**
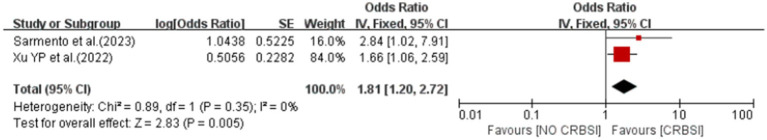
The forest plot shows the relationship between catheterization of the upper and lower limbs and the risk of CRBSI.

A meta-analysis of two studies showed that catheterization of the upper and lower limbs was associated with the risk of CRBSI (OR = 1.81; 95% *CI*: 1.20–2.72; *I^2^* = 0%; Figure 11).

### 3.5 Sensitivity analyses and publication bias

Sensitivity analysis demonstrated the robustness of the pooled results for most risk factors, as the effect estimates remained statistically unchanged upon the sequential removal of individual studies. However, the exclusion of specific studies significantly influenced the heterogeneity estimates for certain factors. Specifically, the substantial heterogeneity observed for the 5-min Apgar score ≤ 7 (*I^2^* = 74%) was markedly reduced (*I^2^* = 9%) after omitting the studies by Li CH et al. (17), Huang CM et al. (18), and Hu Y et al. (23). Similarly, the heterogeneity for the number of manipulations ≥ 2 decreased from 77 to 0% after the removal of Li X et al. (15). The *I^2^* value for catheter indwelling time >14d dropped from 59 to 45% upon excluding Lv Q et al. (16) and Geldenhuys et al. (26), while the heterogeneity for birth weight <1,500 g was reduced from 61 to 35% after omitting the study by Li X et al. (15) (Supplementary material 2).

**Figure 12:**
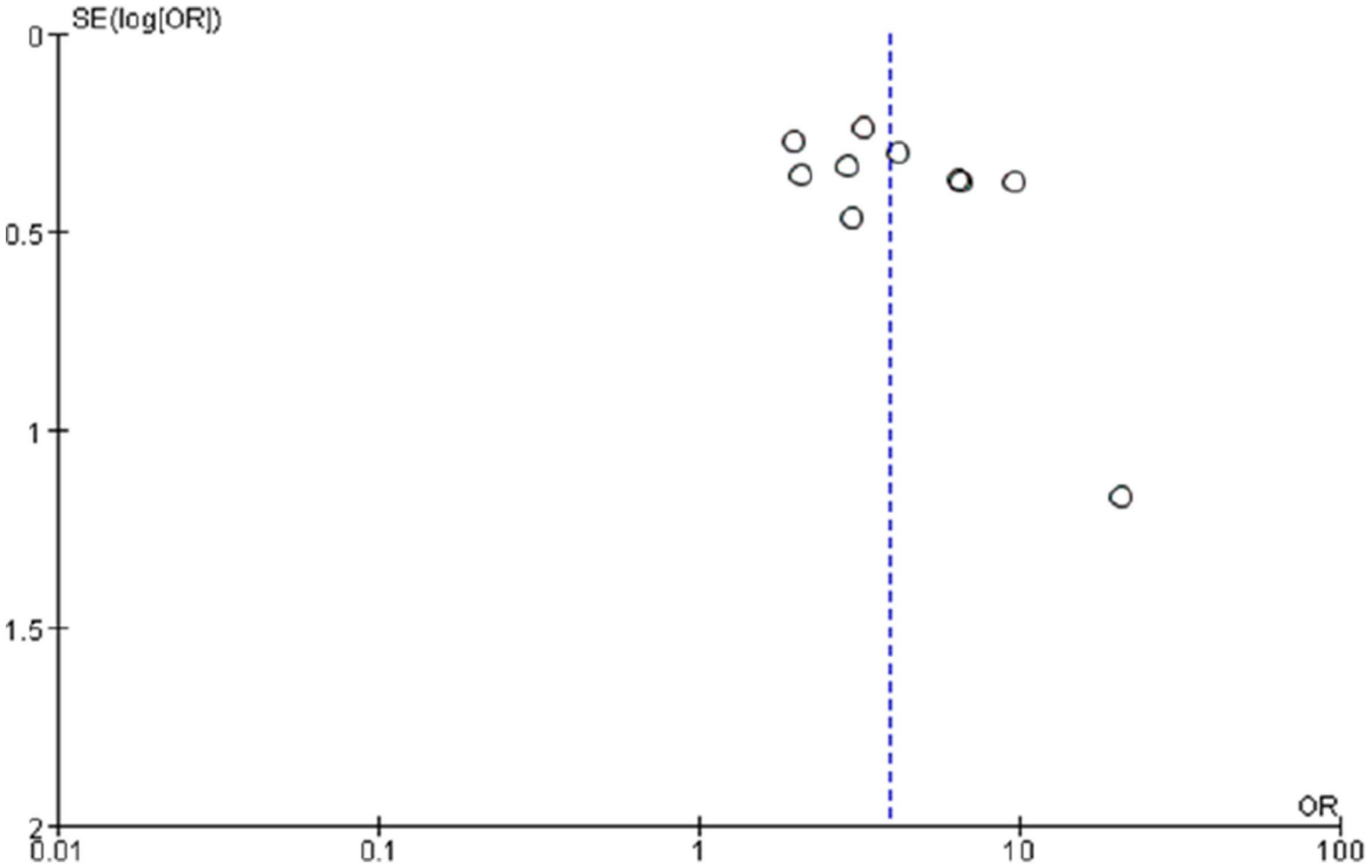
Publication bias funnel plot for catheter indwelling time >14d. Each small circle represents an independent study for the indicated association. The funnel plot appears asymmetric. OR, odds ratio; SE, standard error.

Potential publication bias was assessed for outcomes involving 10 or more studies. The funnel plot and Egger’s regression test indicated no significant publication bias among studies reporting catheter indwelling time >14 d (Egger’s test: *p* = 0.729; Figure 12). For the remaining risk factors, which included fewer than 10 studies, formal statistical assessment of publication bias was not performed, as the power of such tests would be considerably low.

## 4 Discussion

Catheter-Related Bloodstream Infection (CRBSI) has been shown to be significantly associated with prolonged mechanical ventilation, longer ICU and hospitalization stay, and a substantial increase in total hospitalization costs in both domestic and international studies (31–33). In our meta-analysis, we identified 11 risk factors associated with neonatal catheter-related bloodstream infections. It is worth noting that other relevant risk factors have been reported in the literature, such as the use of umbilical vein catheters (UVCs) (34), the use of multiple catheters (35), catheter hub colonization/blood sampling for purposes other than blood gas analysis/catheter exit site disinfection (36), and cesarean delivery (29). Among these, UVCs are a common type of catheter in neonates, but few studies have reported them as a risk factor for CRBSI, which prevented us from including them in our systematic analysis.

Central venous catheters (CVCs) are primarily used for the administration of fluids, vasoactive agents, blood transfusions, and parenteral nutrition. Compared to single-lumen CVCs, multi-lumen CVCs are characterized by a higher number of ports and a corresponding increase in catheter manipulation. These features may provide additional portals of entry for pathogens and create a microenvironment that favors microbial colonization, which is associated with an elevated risk of CRBSI (37–39). Meanwhile, by centralizing nursing procedures and reducing unnecessary opening of catheter access ports, the risk of contamination due to excessive manipulation can be effectively minimized. Generally, the higher the number of punctures, the higher the incidence of CRBSI (13, 17). Repeated puncture (≥2 times) impacts the local subcutaneous tissues and the inner walls of blood vessels in newborns, increasing the risk of bacterial invasion, inflammatory reaction, and infection (40). Our analysis showed that factors such as inadequate preoperative preparation, unskilled catheter placement, and catheter blockage were associated with a significantly higher incidence of CRBSI, a finding consistent with reports from China (41, 42). Therefore, the experience of catheterization operators is a key modifiable factor.

Consistent with previous reports (43, 44), immaturity—reflected by low gestational age and birth weight—compromises innate immunity and skin barrier integrity, predisposing neonates to CRBSI. Our analysis specifically identified gestational age ≤32 weeks and birth weight <1,500 g as key thresholds. This risk profile is further compounded by a 5-min Apgar score ≤7, a marker of physiological depression that is associated with immunodeficiency, reliance on invasive support, and baseline instability, collectively increasing susceptibility to infection (45–47). In addition, a catheter indwelling time for more than 14 days is linked to the incidence of CRBSI. Upon insertion into a blood vessel, catheters become coated with plasma proteins, forming a fibrin sheath. However, the fibrin sheath also facilitates bacterial adhesion. In hosts with competent immunity, adherent bacteria may be cleared before forming a mature biofilm. Conversely, in immunocompromised hosts or when bacteria succeed in establishing a biofilm, they become shielded from host defenses, leading to a high susceptibility to clinical CRBSI. (48). Our findings are supported by Torre et al. Major risk factors for CRBSI in the pediatric intensive care unit include the use of multiple central venous catheters simultaneously and prolonged use of intravenous catheters (49). To reduce the risk of infection, it is advised to closely monitor the indications for catheter insertion and the timing of catheter removal and to limit unnecessary catheter placement. However, the optimal timing for central venous catheter use in neonates remains uncertain. Greenberg et al. recommended replacing central venous catheters within 35 days to reduce the risk of CRBSI (24, 50). A prospective cohort study carried out by Ekaputri et al. demonstrated that PICC dwell times of more than 14 days were associated with a 2.9-fold increase in the risk of CRBSI (2). This appears to be relevant to the pathogenesis of CRBSI, as the generated biofilm can extend into the vasculature within 11 days (25).

The results of a descriptive cohort study by Dubbink et al. showed that male individuals were associated with a higher risk of CRBSI (26). However, the authors noted that causality could not be established because of the observational study’s potential for bias. Furthermore, before complete enteral feeding is achieved, parenteral nutrition is crucial for the care of neonates, particularly premature infants. Parenteral feeding during catheterization, however, raises the risk of CRBSI (26). The lipid content of fluids identified in previous research, such as the multipurpose mixture Minimix, may be connected to this (51). Minimix is a multipurpose liquid lipid-containing mixture. It can be incorporated into enteral feeding formulas and formulated with specific lipid proportions to improve nutrient absorption efficiency. For parenteral nutrition, early enteral feeding should be prioritized, the duration of parenteral nutrition should be shortened, and the formulation and infusion process should be optimized.

Maternal conditions such as pregnancy comorbidities, premature rupture of membranes, diabetes, and meconium-stained amniotic fluid are associated with an increased risk of neonatal catheter-related bloodstream infections (CRBSI), possibly via mechanisms involving neonatal immune compromise (19). Perinatal factors, including maternal comorbidities, fetal status, and intrapartum management, influence Apgar scores and neonatal outcomes (52). Furthermore, Xu YP et al. found that PICCs placed in the head and neck region were associated with lower CRBSI rates compared to those placed in the upper or lower limbs (25). Another systematic review showed no significant difference in catheter-related infection rates between different insertion sites in the upper and lower limbs (53). Although the selection of a catheter insertion site is influenced by the infant’s condition and medical resources, an evidence-based prioritization strategy should be implemented when feasible to avoid sites with higher infection risks.

Based on the findings of this meta-analysis, clinical prevention efforts should focus on the aforementioned modifiable factors. We recommend implementing a bundled intervention strategy, with key components including: (1) strict management of catheter indwelling duration and manipulation frequency; (2) standardized administration of parenteral nutrition; (3) adherence to aseptic techniques and hand hygiene; and (4) optimal selection of catheter type and insertion site. By concentrating limited medical resources on these modifiable factors, a significant reduction in the incidence of CRBSI among neonates—particularly critically ill infants with non-modifiable risk factors—can be anticipated.

This meta-analysis offers a robust synthesis of neonatal CRBSI risk factors, conducted in accordance with PRISMA guidelines and based on a substantial cohort from 18 studies (*n* = 11,963). Nonetheless, several limitations warrant consideration. The statistical power for some risk factors was limited due to the small number of available studies. Although the search strategy was comprehensive, the restriction to specific databases and languages may have introduced selection bias. Furthermore, causal inference is precluded by the observational design of all included studies, while the substantial heterogeneity and the geographic concentration of studies in China may affect the generalizability of some findings.

## 5 Conclusion

Our systematic review and meta-analysis identified several risk factors associated with the development of catheter-related bloodstream infections in neonates. Early detection of risk factors and prevention is the key to reducing the incidence of catheter-related bloodstream infections in neonates. Therefore, systematic surveillance and prevention planning for neonatal catheter-related bloodstream infections should be widely implemented globally. More high-quality randomized studies on neonatal catheter-related bloodstream infections are needed. Such research will further strengthen the evidence base and facilitate the use of findings for evidence-based clinical practice.
