# Supplementary material for: Risk factors for neonatal catheter-related bloodstream infections: a systematic review and meta-analysis
Source: Front Public Health. 2025 Dec 19;13:1719016. doi: 10.3389/fpubh.2025.1719016 (PMC12757328; doi:10.3389/fpubh.2025.1719016)
Supplement: Supplementary file 1 [file Supplementary_file_1.docx]

**The complete search strategy is as follows:**

**1. PubMed database**

Retrieval formula:

("catheter-related bloodstream infection"[Title/Abstract] OR "CRBSI"[Title/Abstract] OR "central line-associated bloodstream infection"[Title/Abstract]) AND ("Neonate"[Title/Abstract] OR "Neonatal"[Title/Abstract] OR "Newborn"[Title/Abstract]) AND ("risk factors"[Title/Abstract])

Time range: From the establishment of the database to October 1, 2025

Language restriction: No restrictions

**2. Web of Science Database**

Retrieval formula:

TS=("catheter-related bloodstream infection" OR "CRBSI" OR "central line-associated bloodstream infection") AND TS=("Neonate" OR "Neonatal" OR "Newborn") AND TS=("risk factors")

Time range: From the establishment of the database to October 1, 2025

Language restriction: No restrictions

**3. Scopus database**

Retrieval formula:

TITLE-ABS-KEY (("catheter-related bloodstream infection" OR "CRBSI" OR "central line-associated bloodstream infection") AND ("Neonate" OR "Neonatal" OR "Newborn") AND ("risk factors"))

Time range: From the establishment of the database to October 1, 2025

Language restriction: No restrictions

**4. China National Knowledge Infrastructure (CNKI)**

Retrieval formula:

Subject = (" Newborn "OR" Premature Infant "OR" Neonatal Period ") AND subject = (" Catheter-related Bloodstream Infection "OR" CRBSI "OR" Central Venous Catheter Infection ") AND subject = (" Risk Factors "OR" Risk Factors ")

Time range: From the establishment of the database to October 1, 2025

Language restriction: No restrictions

**5. Wanfang Database**

Retrieval formula:

(Key words: "Newborn" OR "premature infant") AND (key words: "catheter-related bloodstream infection" OR "CRBSI") AND (key words: "Risk factors")

Time range: From the establishment of the database to October 1, 2025

Language restriction: No restrictions
