# Supplementary material for: Risk factors for neonatal catheter-related bloodstream infections: a systematic review and meta-analysis
Source: Front Public Health. 2025 Dec 19;13:1719016. doi: 10.3389/fpubh.2025.1719016 (PMC12757328; doi:10.3389/fpubh.2025.1719016)
Supplement: Supplementary file 2 [file Supplementary_file_2.docx]

**Supplementary 2**

**Sensitivity analyses**


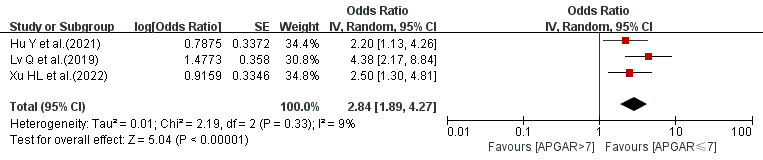


**FIGURE 3**

The forest plot shows the relationship between 5-min Apgar score (APGAR) ≤7 and the risk of CRBSI (adjusting).


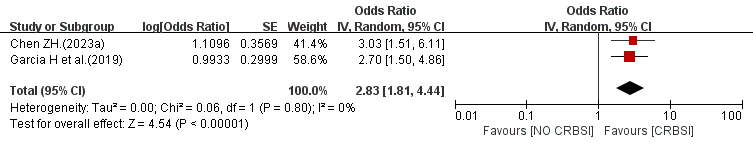


**FIGURE 4**

The forest plot shows the relationship between number of manipulations≥2 and the risk of CRBSI (adjusting).


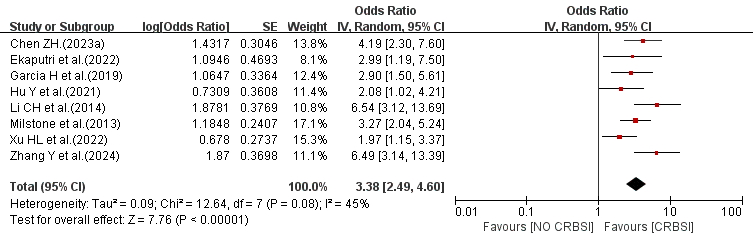


**FIGURE 5**

The forest plot shows the relationship between catheter indwelling time>14d and the risk of CRBSI (adjusting).


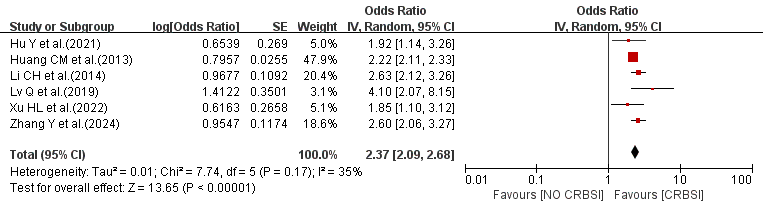


**FIGURE 9**

The forest plot shows the relationship between birth weight<1500g and the risk of CRBSI (adjusting).
